# Introduction to the special issue on small-angle scattering

**DOI:** 10.1107/S160057671601904X

**Published:** 2016-12-01

**Authors:** Michael Gradzielski, Andrew J. Allen

**Affiliations:** a Stranski Laboratorium für Physikalische und Theoretische Chemie, Institut für Chemie, Technische Universität Berlin, Strasse des 17 Juni 124, 10623 Berlin, Germany; b Materials Measurement Science Division, National Institute of Standards and Technology (NIST), 100 Bureau Drive, Gaithersburg, MD 20899, USA

**Keywords:** small-angle scattering

## Abstract

This open-access collection of 11 selected articles covers a small but quite diverse and interesting part of the much wider range of scientific topics presented at the 16th International Conference on Small-Angle Scattering (SAS2015) in Berlin. The topics contained here describe the particular directions in which small-angle scattering is developing at the current moment and which will become increasingly important in the future. The virtual special issue is available at http://journals.iucr.org/special_issues/2016/sas2015/.

The 16th International Conference on Small-Angle Scattering (SAS2015) was held in Berlin, Germany, from the 13 to 18 September 2015 in the main building of the Technische Universität (TU) Berlin, which is centrally located in the city of Berlin, thereby supplying a stimulating environment for this scientific meeting. In preparation for this conference, 424 delegates from 32 countries had submitted 514 abstracts across 12 main themes. Naturally, the largest attendance came from the host country, but the global appeal of this conference was underlined by the fact that attendees from the USA and Japan were the second and third largest groups. The conference Chair was Peter Fratzl from the Max Planck Institute for Colloids and Interfaces, Golm, Germany, assisted by the Co-Chairs, Matthias Ballauff from the Helmholtz Center Berlin (HZB) and Michael Gradzielski from the Technical University of Berlin. Members of the Scientific Program Committee also included Franziska Emmerling, Peter Müller-Buschbaum, Oskar Paris, Anton Plech, Dieter Richter, Peter Schurtenberger, Dmitri Svergun and Regine Willumeit. The hard work of the entire Scientific Program Committee enabled the development of a very interesting conference program encompassing all fields of small-angle scattering (SAS), and this very much formed the foundation for the success of SAS2015.

Setting what we hope will be a new precedent for the SAS conferences, the winners of the Guinier Prize, both in 2012 (Professor Otto Glatter) and in 2015 (Professor Sow-Hsin Chen), were invited to give Plenary Lectures on their lifetime achievements. Each gave an inspirational talk on the development and power of SAS as a method for deducing detailed structural information on mesoscopically structured systems.^1^ For comprehensive information on the conference, including plenary speakers, prize winners, satellite meetings *etc*., the reader is referred to http://www.helmholtz-berlin.de/events/sas/index_en.html.

The culture of publication has seen a significant shift during the past 10–15 years and traditional conference proceedings have become increasingly less popular for publishing results presented at conferences. This is even more true for SAS than for other technique-based conferences, as here, while the method of small-angle scattering is the focus point, many different scientific communities use SAS as a central tool of investigation. Accordingly, many attendees will prefer to publish their best results in journals more specific to peer review in the relevant field. This is increasingly the case as the research fields tackled by SAS have become ever more diverse in recent years. By contrast, we think that this special issue containing a selected number of high-quality peer-reviewed articles, based on work presented at the SAS2015 conference, will prove to be a very valuable source for people working in the broad field of SAS-related science. Naturally, the articles contained within it are somewhat focused towards the SAS technique, itself, and recent developments of the method. For such a topic a natural home is *Journal of Applied Crystallography*, which addresses exactly the clientele interested in this scientific field.

The articles contained in this special issue were subjected to the usual rigorous review process for regular articles submitted to *Journal of Applied Crystallography*. This ensured that only selected high-quality manuscripts are included here. These articles have been published in recent regular issues and are now collected here for the SAS2015 virtual special issue. We are grateful to all the authors that submitted material, whether selected or not, as well as to our Guest Co-editor, Andrew Jackson, and the many referees whose valuable efforts helped to improve the quality of the manuscripts further. All of this made the publication of this special issue possible. We believe this special edition publishes work that provides insights into ongoing developments in the field of small-angle neutron and X-ray scattering (SANS and SAXS) covering different areas of fundamental and applied research.

Lehmkühler *et al.* (2016 ▸) describe the use of X-ray cross-correlation analysis applied to the investigation of colloidal crystals. For the case of poly(methyl methacrylate) colloids it is shown how information beyond the static structure factor can be deduced from coherent X-ray scattering experiments, for example, enabling assignment of a face-centered cubic structure to the crystal. In a different direction, the work of Perkins *et al.* (2016 ▸) dwells on the current state of the atomistic modeling of scattering data and reviews the achievement of the Collaborative Computational Project for Small Angle Scattering (CCP-SAS). Certainly these developments will be important for the future when increasingly complex systems will probably need to be characterized by SAS with atomistic resolution.

The analysis of increasingly complex structured systems is an ongoing development in SAS that will be of key importance in the future. In this area, Oba *et al.* (2016 ▸) studied steel structures by magnetic scattering with simultaneous measurements of small-angle neutron scattering and Bragg-edge scattering. In a related study, Noda *et al.* (2016 ▸) investigated rubbery polymer materials with different inorganic fillers by means of contrast variation using dynamic nuclear polarization. From the measurements with different scattering contrasts one can deduce the partial scattering functions and thereby information not accessible in any other way.

SAS has become increasingly important over the years for the investigation of soft matter systems. An interesting example of such an investigation is given by Prevost *et al.* (2016 ▸), which successfully shows how to obtain detailed structural information on so-called ‘ultra-flexible microemulsions’. This is a novel type of self-assembled system that exhibits microemulsion structures even in the absence of a typical surfactant, as was elucidated here using a combination of SAXS and wide-angle X-ray scattering. Much larger scattering objects were the focus of von Gundlach *et al.* (2016 ▸), who studied whole *Escherichia coli* cells by SAXS and ultra-small-angle X-ray scattering (USAXS) to cover the full relevant scale range. This work allowed a comprehensive model to be deduced that is based on the main structural cell components, *i.e.* ribosomes, DNA and proteins. These authors also investigated the effect of different antibiotics on this collective structure. By contrast, synthetic systems can also be of high scientific interest, as demonstrated by Kaneko *et al.* (2016 ▸) for the case of syndiotactic polystyrene cocrystals with polyethylene glycol dimethyl ether. The temperature-dependent changes from crystalline to more amorphous structures were obtained by combining SANS investigations with simultaneous Fourier transform infrared spectroscopy measurements.

Another way of coupling SAS experiments with complementary information is shown by Jordan *et al.* (2016 ▸), who demonstrate how size exclusion chromatography (SEC) can be coupled to SANS experiments. This development relies on the fact that, owing to increasing available neutron flux, investigation of small volumes in sufficiently short time intervals (30 s) has become possible. In a proof-of-principle experiment the authors show how different proteins in a mixture can become separated by SEC and then can be mesoscopically characterized by SANS. This development may mark the start of increasing use of SANS to study the composition of more complex colloidal mixtures. Instrumentation is also the focus of Li *et al.* (2016 ▸), who report on the state of the BioSAXS beamline BL19U2 at the National Center for Protein Sciences Shanghai. This new synchrotron SAXS beamline is dedicated to meeting the increasing demands of researchers from the field of structural biology and is optimized for delivering a large dynamic scattering vector range.

Finally the field of grazing-incidence SAXS (GISAXS) has also seen substantial development in recent years. Here, this is demonstrated by the work by Fritz-Popovski *et al.* (2016 ▸), which used GISAXS to investigate mesoporous ordered silica films obtained by templating a copolymer. These experiments allowed information to be deduced on the pore structure and orientation in these films, and the effects of water sorption into these films could be followed. Another fine example of this technique and its potentials is given by the investigation of the effect of swift heavy ions on TiO_2_ films. The effect of ion impact on rutile surfaces was studied by GISAXS by Karlušić *et al.* (2016 ▸), and valuable information about the surface track as a function of irradiation flux could be deduced.

With this collection of 11 selected articles we cover a small but quite diverse and interesting part of the much wider range of scientific topics presented at SAS2015 in Berlin. The topics contained here describe the particular directions in which SAS is developing at the current moment and which will become increasingly important in the future. The issue is fully open access and is available at http://journals.iucr.org/special_issues/2016/sas2015/.

Of course, SAS continues to develop in all its forms, and the next conference, SAS2018, will be held in Traverse City, Michigan, USA. We wish the organizers of this meeting every success. For sure, there will be further substantial advances of the SAS technique, itself, and its application to solve important scientific questions in diverse research areas that will be presented there.

## Footnotes

1 The conference organizers would like to thank the International Union of Crystallography (IUCr) for supporting the Guinier Prize, and especially the IUCr Commission on SAS, chaired by Professor Jill Trewhella.
